# Identifying Barriers to the Adoption of Digital Contact Tracing Apps in England: Semistructured Interview Study With Professionals Involved in the Pandemic Response

**DOI:** 10.2196/56000

**Published:** 2024-08-12

**Authors:** Anna Palmer, Shaishab Sharma, Jayesh Nagpal, Victor Kimani, Florence Mai, Zara Ahmed

**Affiliations:** 1 Imperial College School of Medicine Imperial College London London United Kingdom; 2 Barts and the London School of Medicine and Dentistry Queen Mary University of London London United Kingdom; 3 Birmingham Medical School University of Birmingham Birmingham United Kingdom; 4 Imperial College Business School Imperial College London London United Kingdom

**Keywords:** COVID-19, global health, public health, qualitative study, tracing, England, apps, effectiveness, contact tracing, barrier, digital health, thematic analysis, privacy, communication, social support, tracing app, digital illiteracy, technology, support

## Abstract

**Background:**

The NHS (National Health Service) COVID-19 app was a digital contact tracing app (DCTA) used in England in response to the COVID-19 pandemic. The aim of which was to limit the spread of COVID-19 by providing exposure alerts. At the time of the pandemic, questions were raised regarding the effectiveness and cost of the NHS COVID-19 app and whether DCTAs have a role in future pandemics.

**Objective:**

This study aims to explore key barriers to DCTAs in England during the COVID-19 pandemic.

**Methods:**

This is a qualitative study using semistructured video interviews conducted with professionals in public health, digital health, clinicians, health care law, and health executives who had an active role in the COVID-19 pandemic. These interviews aimed to explore the perspective of different experts involved in the pandemic response and gauge their opinions on the key barriers to DCTAs in England during the COVID-19 pandemic. The initial use of maximum variation sampling combined with a snowball sampling approach ensured diversity within the cohort of interviewees. Interview transcripts were then analyzed using Braun and Clarke's 6 steps for thematic analysis.

**Results:**

Key themes that acted as barriers to DCTAs were revealed by interviewees such as privacy concerns, poor communication, technological accessibility, digital literacy, and incorrect use of the NHS COVID-19 app. Interviewees believed that some of these issues stemmed from poor governmental communication and a lack of transparency regarding how the NHS COVID-19 app worked, resulting in decreased public trust. Moreover, interviewees highlighted that a lack of social support integration within the NHS COVID-19 app and delayed app notification period also contributed to the poor adoption rates.

**Conclusions:**

Qualitative findings from interviews highlighted barriers to the NHS COVID-19 app, which can be applied to DCTAs more widely and highlight some important implications for the future use of DCTAS. There was no consensus among interviewees as to whether the NHS COVID-19 app was a success; however, all interviewees provided recommendations for improvements in creating and implementing DCTAs in the future.

## Introduction

After the first UK lockdown in March 2020, there was strong motivation for an effective test and trace system to help curb the spread of COVID-19 because at this point the United Kingdom recorded one of the highest confirmed death rates worldwide in 2020 [1].

Launched on September 24, 2020, the NHS (National Health Service) COVID-19 app was England’s response to curb the spread of COVID-19. £76 million of the £47 billion (exchange rate at time of the study was US $1.24) ringfenced COVID-19 funding was spent on development, with the investment justified on the premise that it would avert another lockdown. Yet, 2 more lockdowns followed, and questions were raised regarding the effectiveness of such digital contact tracing apps (DCTAs) and their use in future pandemics [2-4].

It was believed that the transmission of COVID-19 could be reduced by an anonymized contact tracing app, which could rapidly deliver exposure notifications [5], thus the NHS COVID-19 app was created. This app was endorsed by the UK government as a public health intervention that would digitally contact trace individuals exposed to COVID-19 and request that they self-isolate to prevent further virus spread [6]. The NHS app used a decentralized framework, which stored data regarding the proximity of contacts through “Low Energy Bluetooth” signals and created alerts based on a privacy-preserving Google-Apple Exposure Notification System [7]. It aimed to reduce disease spread and achieve pandemic control through exposure alerts. A digital approach increases the speed and accuracy with which it can identify and warn contacts but carries the risk of false positives [8]. Modeling suggested that to achieve pandemic control with no other restrictions, an app adoption rate of 60% of the population was needed [9]. This can be defined by both downloading (uptake) and adhering to the app (correct use and following notifications). In reality, by December 2020, the NHS COVID-19 app had just 16.5 million users, accounting for 28% of the population in England and Wales [10]. Thus, this research aims to examine why this may be the case by focusing on the barriers to the adoption of DCTAs through semistructured interviews (SSIs) with experts, aiming to answer the specific question: What were the key barriers to the adoption of DCTAs in England?

## Methods

### Study Design

This qualitative study consisted of SSIs, which were carried out from a period between March 7, 2022, and April 14, 2022. All interviewees were asked the same main questions, allowing for consistency across interviews. These interviews aimed to explore the perspective of different experts involved in the pandemic response and gauge their opinions on practical solutions that can be used to improve the implementation of DCTAs in the future. The results were recorded and then thematically analyzed. SSIs were preferred over focus groups since SSIs avoid groupthink and ensure sufficient time for all participants’ individual thoughts to be explored [11].

### Recruitment

A range of interviewees with different expertise was desired to ensure a broader input into the study; therefore, prospective interviewees were identified using Google and social media searches for job descriptions, who were then contacted via email or LinkedIn to request and arrange an interview. Initially, maximum variation sampling [12] was attempted with 6 interviewees recruited via this method but due to time constraints and difficulties securing a large enough sample, a snowball sampling approach was used, with interviewees asked to recommend other experts who may be useful to speak to. Adopting a snowball sampling method can over time skew the sample to 1 type of professional; however, maximum variation sampling to identify the first participants mitigated the effect [13]. No exclusion criteria were applied; however, inclusion criteria included the ability to conduct Microsoft Teams meetings, involvement in COVID-19 response, and being situated in England and able to speak English.

Recruitment ended after 12 interviews as thematic saturation was achieved [14]. Interviewees included public health experts, health app developers, and clinicians. Information about the study was communicated to participants in the initial contact email and reiterated at the beginning of the interview. Prior to conducting the interviews, consent for recording and transcription through Microsoft Teams was obtained via a consent form and was obtained again verbally at the beginning of each interview (Multimedia Appendix 1)*.* All interviewees were given the right to view their transcription and withdraw from the study.

### Procedure

The SSIs followed a predetermined interview guide, with follow-up questions permitted. Open-ended questions were asked to facilitate detailed responses. A complete interview guide can be found in Multimedia Appendix 2*.* Questions covered the NHS COVID-19 app, NHS test and trace, DCTAs more generally, other European countries’ implementations of contact tracing, and improvements for the future. The 2 pilot interviews were conducted by the same 2 individuals who undertook all interviews. The duration of the interviews was intended to be 60 minutes. To ensure research trustworthiness, the criteria of credibility, transferability, dependability, and confirmability of Lincoln and Guba [15] were followed throughout.

### Ethical Considerations

This study was exempted by the Imperial College Research Ethics Committee and Science, Engineering and Technology Research Ethics Committee. Informed written consent was obtained prior to the interview and was obtained again verbally at the beginning of each interview. The interviewees in this study have been anonymized, with only profession being listed to provide context to the results. No compensation was provided to interviewees.

### Data Analysis

Interview results were analyzed using Braun and Clarke's 6 steps for thematic analysis [16]. One of the researchers transcribed all interviews verbatim based on the transcriptions provided by Microsoft Teams and the recorded audio files of the interviews. Then, 3 coders independently coded 3 of the transcripts to create a preliminary codebook, which was afterward discussed, and agreements were made on code definitions, to ensure consistency in the application of the codebook.

**Figure 1:**
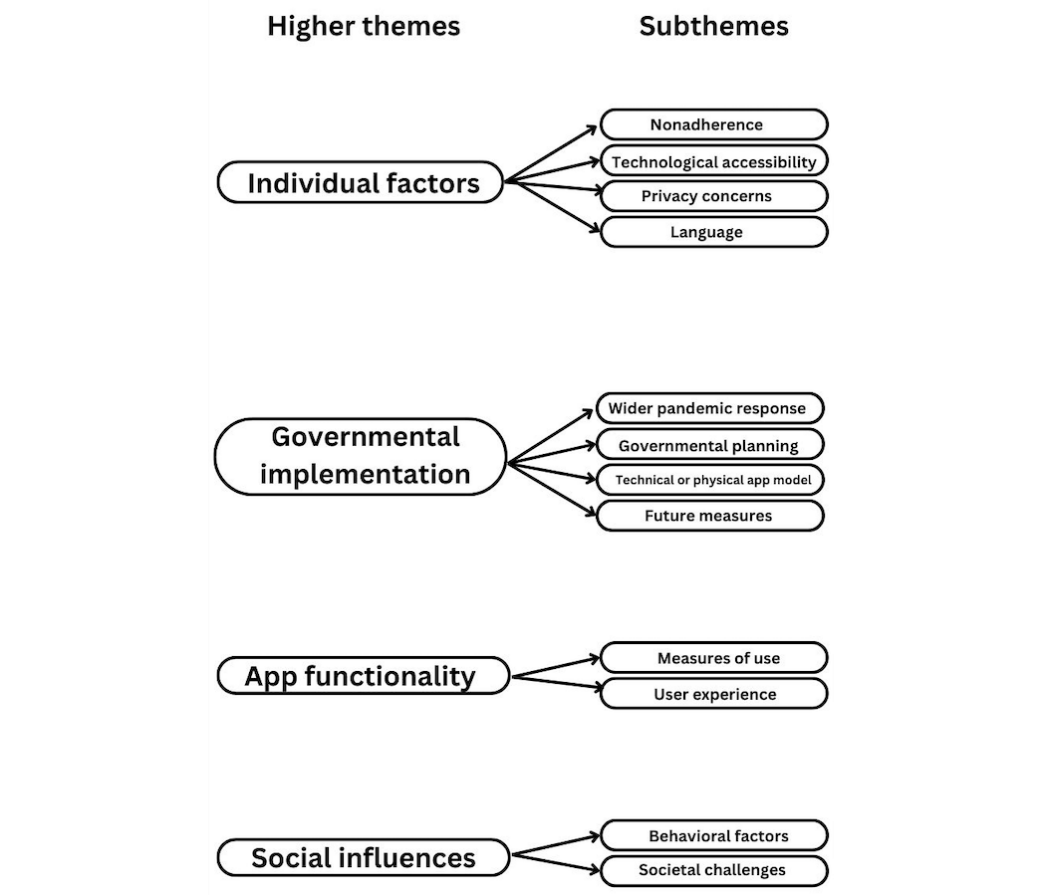
Thematic analysis.

Coders independently applied the codebook to the remaining 9 interviews and generated additional codes for the codebook. Any disagreements over codes were settled by a third independent coder. A thematic map was produced, with codes arranged into higher themes and subthemes (Figure 1).

As indicated by the step of theme reviewing, coding is a continual process. As new codes were added, themes were continually critiqued to ensure relevance and encompass the full essence of the data [16]. Codes found to be irrelevant were removed, and codes that had initially been omitted were added back to the themes. The coders then selected key quotes from the data set to illustrate the codes in their reporting.

## Results

### Interviews

This study included interviews with 12 experts, grouped by their occupation: 3 experts in the digital health field, 5 in public health, 2 clinicians, 1 executive in health care law, and 1 health executive at a large health care trust. Analysis of the data uncovered 4 higher themes, each containing multiple subthemes. This is represented by the thematic map (Figure 1). Of the 12 experts, 9 experts were male and 3 female, other demographics were not collected. The duration of the interviews ranged from 20 to 82 minutes, with an average time of 44 minutes.

### Individual Factors

When considering individual factors which hindered the effective use of the NHS COVID-19 app, interviewees made references to privacy concerns and the resulting indecision regarding a centralized versus decentralized system which led to “scepticism from the start around some of the [kind of] privacy considerations” [Public health expert 2]. Similarly, a lack of trust in the app was raised by 8 interviewees, as effective DCTAs “require trust and confidence in the owner of the technology” [Clinician 1].

A lack of clear communication resulted in public confusion over the app’s functionality, with half of interviewees suggesting the NHS COVID-19 app would have benefited from increased transparency regarding data governance: “Lack of public awareness within the population is probably a factor” [Public health expert 1]. Another interviewee believed that a lack of communication causes a lack of understanding and that it is important *“*to start by making sure everyone understands how contact tracing works more generally” [Digital health expert 2].

Nonadherence was another issue raised by 3 interviewees as, in particular, the ability to “turn off” the NHS COVID-19 app, 1 interviewee said: “People intentionally would turn their Bluetooth off” [Digital health expert 3].

During interviews, questions surrounding device accessibility, digital illiteracy, and user accessibility were raised: “I have 70-year-olds walk into my clinic who have a Nokia 310 ... if you don't have the ability to use that, you're never going to be able to reach that part of the population” [Clinician 2].

The NHS COVID-19 app is “assuming a level of technology. It's assuming a level of access to the Internet ... the digital capability of the individual using it” [Clinician 1]. Interviewees questioned whether the app catered to those with limited ability to understand English and if accessibility could be increased by *“*having different languages, having different ways of understanding it*”* [Digital health expert 3].

### Governmental Implementation

Among the interviewees, there were conflicting ideas about the purpose of the NHS COVID-19 app*,* one interviewee believed *“*the app was best at building trust for population” [Digital health expert 1] while another felt “the purpose was to prevent deaths, prevent lockdowns” [Public health expert 1].

Virtually all interviewees questioned if the government made the correct strategic decisions when implementing the app: “there were different people saying different things and a lack of leadership” [Public health expert 3] and 1 interviewee believed “there was not a strategy” [Clinician 1].

Another issue raised by interviewees was the cost of the app: “It could have been spent elsewhere. It was a huge amount of money” [Health executive 1].

Many had suggestions on how it could be improved.

I would have preferred it if public health officials had more direct control ... had a greater say on how it could be repurposed for the future ... that is a weak point of the current system.

Digital health expert 3

There was no clear consensus among interviewees as to whether DCTA may be useful in future pandemics as “it depends on the characteristics of the pandemic” [Clinician 1] and “the nuances of why the contact tracing app is helpful ... is to do with the dynamics of COVID-19 in particular” [Digital health expert 3].

The need for better preparation for the next pandemic was emphasized by interviewees who said DCTAs need to be “already in place at the beginning” [Public health expert 5] with the potential to “tailor to a specific virus or pathogen” [Public health expert 2]. Multiple interviewees suggested this could be made possible by encouraging installation prior to a pandemic, for example, having an app on standby.

Other suggestions for improvement included increasing connection between stakeholders to keep the “evidence base up to date in some way” [Digital health expert 3]*.* Alongside improved integration: “Close integration with the testing system is important” [Public health expert 4]. Clear messaging was another factor mentioned by interviewees as “you need to go to your target audiences, and you need to disseminate your message” [Public health expert 1].

### App Functionality

The lack of functional uses of the NHS COVID-19 app and its separation from the NHS app debated “it doesn’t make a lot of sense to have two apps on your phone” [Health executive 1]. There was a desire expressed by interviewees for a more integrated approach as “the main app is just a lot of links” [Digital health expert 1].

The delayed notification period of the app was noted as a disadvantage since it decreased the effectiveness of transmission control: “You’re walking around positive for days before you would actually be able to send that notification up to others” [Digital health expert 3].

Interviewees were also concerned by the reduction in app interest, despite initial high uptake, one suggested that measuring app installations was fundamentally flawed: “I think success is rarely someone installing it on their phone and leaving it on their phone” [Digital health expert 1]*.* One of the interviewees mentioned that “you need to be really careful that you don’t ever get to that tipping point where people start ignoring the messages that they’re getting from it” [Digital health expert 2].

Some interviewees were concerned by the ability of the app to recognize genuine contacts of positive cases, and its inability to take occupation into context: “It was just pinging ... clearly it was not up to scratch in terms of its technical ability” [Clinician 2].

Other interviewees were positive about the function of the app and believed that “the app we ended up with was the right app to have” [Digital health expert 3] and therefore, “the British Isles had good digital contact tracing” [Public health expert 5].

### Social Influences

The influence of culture on the use of the NHS COVID-19 app was discussed by 3 interviewees*,* and in particular, whether “tracking” people is deemed to be socially acceptable: “We would never have accepted an app that tracked us everywhere we went,” [Digital health expert 2]. Another believed that the altruism required for successful use was an issue: “One of the biggest challenges is you’re still asking someone to do a thing where there is no immediate benefit or reward” [Digital health expert 1].

Interviewees felt that access to social support should be integrated into the app to increase the proportion of people who adhered to self-isolation requests: “After notification, you need to give people clear information and clear support” [Public health expert 5].

Concerns regarding the financial and social cost of self-isolation were noted as a potential societal barrier.

If people are self-isolating, what’s their home environment like? ... it’s really difficult for their mental health.

Public health expert 2

Emphasizing that the financial cost of self-isolation was a significant barrier for those on “low income, zero hours contracts or without much job security” [Public health expert 2].

## Discussion

### Principal Findings

Views from 12 experts in 5 different fields relevant to the pandemic response were sought to explore the key barriers to the adoption of the NHS COVID-19 app. Many experts cited the same themes as barriers to the adoption of the NHS COVID-19 app such as trust, nonadherence, technological accessibility, the strategy, and implementation of the app as well as social factors that hindered the effective use of the app.

More than half of the experts interviewed cited technological accessibility as a barrier to app adoption; within the United Kingdom, there is widespread inequality regarding internet and technology access which has been exacerbated by COVID-19 [17,18]. Similarly, digital literacy was cited as a barrier; in the United Kingdom, there are 11.3 million people who lack basic digital skills and 4.8 million people who do not access the internet at all [19]. Interviewees highlighted that access to a smartphone device should not be assumed and further efforts to improve digital literacy in the community should occur. Accessibility is also important with regard to other aspects of the app as interviewees raised language as an important barrier to effective use of the NHS COVID-19 app as it is only available in 12 languages, including English [20].

Given the limited public understanding and the many conspiracy theories circulating at the time, it is not surprising that privacy concerns were reported by two-thirds of interviewees. This aligns with the literature, where privacy concerns were the most cited reason why people were reluctant to download DTCAs [21-23]. Privacy concerns likely stem from a deeper lack of trust in the government, with studies finding that countries with greater trust in the government had higher rates of app adoption [24]. Since governmental trust levels decreased after the app’s release, this may indicate why prepandemic intentions to download the apps were higher than true adoption rates [25]. Furthermore, NHS COVID-19 app messaging was seen as insufficient by interviewees; it was felt that inconsistent and unclear messaging led to reduced app uptake. Transparency is important in public health interventions, interviewees in the public health field cited that statistics were not being released and there was a general lack of transparency [26]. This study highlights that for DCTAs to be successful in the future, communication from government officials needs to be clear, concise, and transparent. Enabling greater public understanding is likely to increase trust in both the DCTAs and their potential for preventing the spread of a pandemic.

A key feature of the NHS COVID-19 app was the contact tracing notification system, and the resulting self-isolation, if necessary. However, this research highlights that social and financial factors can limit the effectiveness of DCTAs. A common theme was the lack of support for self-isolation which aligns with literature that reports economic necessity and the inability to miss work as key reasons for self-isolation and nonadherence [27]. The United Kingdom provided much less income protection to employees than other countries during the pandemic [28]. Moreover, the £500 support package for low-income workers to self-isolate only covered 1 in 8 workers [29,30]. Participants reported that self-isolation also has a social cost as it is linked to many mental health conditions including depression, anxiety, and other detrimental health effects [31].

### Implications for Practice and Research

This paper provides important insights into the barriers to DCTAs, which are relevant to policy makers and those involved in future pandemic responses. This study highlights that multiple factors contributed to the low uptake and adherence of DCTAs which need to be addressed to improve future use.

This study adds to the literature surrounding DCTAs and adds context to quantitative data surrounding DCTA adoption rates. Future research should aim to include more stakeholder groups and the general population to gather a broader range of views.

### Strengths and Limitations

The nonrandom recruitment of participants based on purposive sampling techniques could lead to the introduction of selection bias in the results gathered. Although participants were selected opportunistically to reach thematic saturation, the insufficient representation of primary care doctors and tertiary care consultants limits the transferability of conclusions. Time constraints resulted in only 12 interviewees, as interviewee recruitment was a time-consuming process; however, a broad range of expertise is covered by these 12 interviewees.

A pilot study was carried out to identify the phrasing of the questions. While care was taken to ensure the questions were phrased neutrally, on reflection the questions could have been improved. While conducting the survey, questions were provided to all interviewees prior to reduce recall bias and ensure fairness. However, this could lead to some interviewees preparing for longer than others. The nature of the SSIs means that questions that were asked varied between interviewees. While this meant that new information was explored in areas of expertise to the participant, the questions were different for each participant leading to bias.

### Conclusions

This research highlights key barriers to the adoption of the NHS COVID-19 app, which hindered its effective use due to decreased public confidence and trust in the NHS COVID-19 app and the government. There was no consensus between experts as to whether the NHS COVID-19 app was a success; however, all experts provided recommendations for future improvements. Social support integrated into DCTAs, to aid those self-isolating and the ability to have a DCTA on standby, ready to be immediately rolled out nationally in future pandemics were the key recommendations from interviewees. This study highlights some important implications for the future use of DCTAs. Although these apps can have great public health benefits, a considerable amount of work needs to be done to ensure that their potential will be maximized in the future.

## Appendix

Multimedia Appendix 1 Consent form.

Multimedia Appendix 2 Interview Questions.

## Abbreviations

DCTA: digital contract tracing app
NHS: National Health Service
SSI: semistructured interview

## Data Availability

Data sharing is not applicable to this article as no data sets were generated or analyzed during this study.
