# Supplementary material for: Identifying Barriers to the Adoption of Digital Contact Tracing Apps in England: Semistructured Interview Study With Professionals Involved in the Pandemic Response
Source: JMIR Form Res. 2024 Aug 12;8:e56000. doi: 10.2196/56000 (PMC11347901; doi:10.2196/56000)
Supplement: Multimedia Appendix 1 [file formative_v8i1e56000_app1.pdf]

Interview Consent Form

Research project title: Overcoming Barriers to the Adoption and Use of Digital Contact Tracing Apps – Lessons Learnt from UK, Germany and Italy during COVID-19

Research Participants Name:

We would like to thank you for agreeing to be interviewed as part of our research project, titled above. Ethical procedures for academic research undertaken from UK institutions require that interviewees explicitly agree to being interviewed and are told how the information contained in their interview will be used. This consent form is necessary for us to ensure that you understand the purpose of your involvement and that you agree to the conditions of your participation. Please, would you therefore read this and the accompanying Participation Information Sheet and then sign this form to certify that you approve the following:

- The audio of the interview will be recorded, and a transcript will be produced
- You will be sent the transcript and given the opportunity to correct any factual errors
- The transcript of the interview will then be analysed by our team as research investigators
- The audio recording will be destroyed after the transcript has been returned
- Any variation of the conditions above will only occur with your further explicit approval

Quotation Agreement

I understand that my words may be quoted directly. With regards to being quoted, please state next to any of the statements that you do not agree with a cross:

- I wish to review the notes, transcripts, or other data collected during the research pertaining to my participation.
- I agree to be quoted directly.
- I agree to be quoted directly if my name is not published and a made-up name (pseudonym) is used.
- I agree that the researchers may publish documents that contain quotations by me.

By signing this form, I agree that;

1. I am voluntarily taking part in this project. I understand that I don't have to take part, and I can stop the interview at any time;
2. The transcribed interview or extracts from it may be used as described above;
3. I have read the Information sheet;
4. I don't expect to receive any benefit or payment for my participation;
5. I can request a copy of the transcript of my interview and may make edits I feel necessary to ensure the effectiveness of any agreement made about confidentiality;

6. I have been able to ask any questions I might have, and I understand that I am free to contact the researcher with any questions I may have in the future.

Printed Name: ______________________________________

Participants Signature: ______________________________________

Date: ______________________________________
