# Supplementary material for: Identifying Barriers to the Adoption of Digital Contact Tracing Apps in England: Semistructured Interview Study With Professionals Involved in the Pandemic Response
Source: JMIR Form Res. 2024 Aug 12;8:e56000. doi: 10.2196/56000 (PMC11347901; doi:10.2196/56000)
Supplement: Multimedia Appendix 2 [file formative_v8i1e56000_app2.pdf]

Supplementary File 2: Interview Questions

1. How would you define failure and success of test and trace?
2. How would you measure success and failure?
3. Which European country had the best system or implemented it the best?
4. In your opinion, what were the major reasons for app failure?
5. How do you think these can be improved on?
6. What are the key characteristics of an effective system?
7. In the future, how would you roll out test and trace?
